# Automated SNP detection from a large collection of white spruce expressed sequences: contributing factors and approaches for the categorization of SNPs

**DOI:** 10.1186/1471-2164-7-174

**Published:** 2006-07-06

**Authors:** Nathalie Pavy, Lee S Parsons, Charles Paule, John MacKay, Jean Bousquet

**Affiliations:** 1 ARBOREA and Canada Research Chair in Forest Genomics, Pavillon Charles-Eugène-Marchand, Université Laval, Ste.Foy, Québec G1K 7P4, Canada; 2 Center for Computational Genomics and Bioinformatics, University of Minnesota, 420 Delaware St. S.E., MMC 43, Minneapolis, MN 55455, USA

## Abstract

**Background:**

High-throughput genotyping technologies represent a highly efficient way to accelerate genetic mapping and enable association studies. As a first step toward this goal, we aimed to develop a resource of candidate Single Nucleotide Polymorphisms (SNP) in white spruce (*Picea glauca *[Moench] Voss), a softwood tree of major economic importance.

**Results:**

A white spruce SNP resource encompassing 12,264 SNPs was constructed from a set of 6,459 contigs derived from Expressed Sequence Tags (EST) and by using the bayesian-based statistical software *PolyBayes*. Several parameters influencing the SNP prediction were analysed including the *a priori *expected polymorphism, the probability score (P_SNP_), and the contig depth and length. SNP detection in 3' and 5' reads from the same clones revealed a level of inconsistency between overlapping sequences as low as 1%. A subset of 245 predicted SNPs were verified through the independent resequencing of genomic DNA of a genotype also used to prepare cDNA libraries. The validation rate reached a maximum of 85% for SNPs predicted with either P_SNP _≥ 0.95 or ≥ 0.99. A total of 9,310 SNPs were detected by using P_SNP _≥ 0.95 as a criterion. The SNPs were distributed among 3,590 contigs encompassing an array of broad functional categories, with an overall frequency of 1 SNP per 700 nucleotide sites. Experimental and statistical approaches were used to evaluate the proportion of paralogous SNPs, with estimates in the range of 8 to 12%. The 3,789 coding SNPs identified through coding region annotation and ORF prediction, were distributed into 39% nonsynonymous and 61% synonymous substitutions. Overall, there were 0.9 SNP per 1,000 nonsynonymous sites and 5.2 SNPs per 1,000 synonymous sites, for a genome-wide nonsynonymous to synonymous substitution rate ratio (*Ka/Ks*) of 0.17.

**Conclusion:**

We integrated the SNP data in the ForestTreeDB database along with functional annotations to provide a tool facilitating the choice of candidate genes for mapping purposes or association studies.

## Background

Large-scale sequencing projects offer a possibility for low cost SNP discovery since sequence variants can be found computationally by analysing the redundancy in sequence databases. For example, this approach has facilitated SNP-based genetic mapping in human [1,2], *Arabidopsis *[3-5], and rice [6,7]. Following the development of pipelines to handle data derived from sequencing platforms, computational tools have been developed to predict SNPs in overlapping fragments of genomic sequences or in expressed sequences such as ESTs [8,9].

The first-generation SNP detection software was derived from tools used to help processing sequence data. After the clustering and alignment of ESTs with *Phrap*, false positives were avoided by applying several filters to remove low quality sequences [10]. Such approaches were applied to public EST databases to identify candidate SNPs, which were verified experimentally in human populations [10]. A major improvement of this approach was the detection and filtering of putative paralogous sequences misassembled into the same cluster. Such filtering was conducted using phylogenetic analysis [11] or sequence similarity searches [12]. Indeed, one difficulty in searching for intraspecific orthologous SNPs in sequence databases is to distinguish true polymorphisms from artifacts due to transcription/sequencing errors or misassembly of paralogous sequences. Without sequence trace data, a confidence score can be deduced from the redundancy at the SNP locus and from its co-segregation with other surrounding SNPs in the alignment [9,13]. Other methods use sequence quality information derived from the raw trace data. Bayesian statistics were applied to incorporate background information into the specification of a tested model for data analysis [14]. They were implemented in the software *PolyBayes *to determine a confidence score for each SNP detected in a cluster of ESTs [15]. *PolyBayes *uses *a priori *information about the average pairwise difference between paralogous sequences to calculate *a posteriori*, the probability that a sequence is native by comparison to a reference sequence from the EST cluster. Thus, sequences classified as paralogous in one EST cluster are excluded from the SNP detection procedure. Then, based on the alignment of the ESTs within the cluster, another Bayesian calculation generates the probability that a variant at a given location of a multiple alignment represents a true polymorphism as opposed to a sequencing error. This calculation takes into account the alignment depth, the base calls in each of the sequences, the associated base quality values, the base composition in the region, and the expected *a priori *rate of polymorphism of the species under investigation. This approach was shown to be adequate for SNP prediction in human [15], sugarcane [16], soybean [17], and pine [18].

The development of SNP markers in plant species will contribute to the understanding of crop evolutionary history, to the positional cloning of QTLs, and to marker-assisted breeding (reviewed in [19]). Especially in conifers, candidate gene approaches are favored to dissect complex traits since linkage desequilibrium is low or declines rapidly within the length of an average-sized gene [20,21]. In these species, the development of high density gene maps based on intraspecific SNPs should result in better marker-aided selection systems by enabling the efficient co-localization of genes and quantitative/qualitative trait loci to further facilitate association studies. These high-density gene maps will also be helpful to delineate homologous chromosomal regions among taxa and to study how gene regions are structured and have evolved (e.g. [22]). To accelerate mapping projects in conifers, we plan to use high-throughput genotyping technologies. A first step toward this goal is the development of a resource of candidate SNPs. Such resources can be obtained computationally since several EST collections are being developed in pines [23-26] and in spruces [27,28].

As part of our research integrating functional and structural genomics analyses in white spruce (*Picea glauca*) [29], we have generated diverse cDNA libraries from 12 genotypes and carried large-scale EST sequencing [30]. Here, we present the *in silico *detection of SNPs in the EST alignments using the *PolyBayes *software and describe the factors that affect the automated SNP detection. The quality of the *in silico *approach was further assessed by the independent sequencing of a subset of genomic DNA fragments. We demonstrate how this analysis was used to define a set of highly reliable predicted SNPs according to their probability scores. Contigs may also contain paralogous sequences which may cause the detection of non allelic SNPs. Parameters for limiting this bias can be set in the prediction software *PolyBayes*, but methods for *a posteriori *assessment of this source of variation remain to be defined. We took advantage of our SNP dataset to evaluate the proportion of paralogous SNPs in our white spruce gene index using statistical and experimental approaches. Two general approaches were also applied to delineate translated regions and distinguish synonymous from nonsynonymous SNPs. To facilitate the choice of candidate genes to be mapped, this white spruce SNP resource was integrated into a publicly available database ForestTreeDB, which also includes functional annotations of the gene index.

## Results and discussion

We estimated SNP diversity and distribution parameters in 6,459 contigs each derived from sequences of at least two cDNA clones with the *PolyBayes *software. Predicted SNP analyses have frequently been conducted based on a number of aligned sequence reads. We have conducted large-scale EST sequencing with both 3' and 5' reads from the same cDNA clones and obtained a large number of cDNAs with of overlapping reads in the same contig [30]. We thus considered the contigs for inclusion in our analysis based upon the number of clones represented rather than the number of reads.

### Experimental error rate within EST assemblies

We exploited the fact that sequence reads in opposite directions from the same clone overlapped in 4,395 contigs, in order to verify whether SNPs were detected in these redundant sequences. Indeed, differences detected within same-clone overlaps would not represent real SNPs but experimental errors from reverse transcription or sequencing. The total available alignment length of these overlapping sequences was 4,755,154 nucleotides. In total, there were only 288 positions (1 per 16,511 nucleotide sites) where reads from the same clone conflicted, and 74% of these positions were uncalled or ambiguous bases. Among the remaining 75 putative false substitutions, we observed a few cases where the discrepancy in one clone was repeated in another clone at the same site in the contig. After removing these repeated discrepancies, we detected 69 sites that conflicted between overlapping reads from the same clone, out of the overall 7,555 SNPs detected with probability score P_SNP _≥ 0.10. The overlapping sequences represented an error rate or false prediction rate of less than 1%. The low level of false prediction indicates that the sequences were generated with a high accuracy and that the initial filtering using a *Phred *score of 20 appears to be sufficiently stringent to eliminate the majority of experimental errors.

### *In silico *detected SNPs according to the expected SNP frequency and PolyBayes probability scores

**Figure 1:**
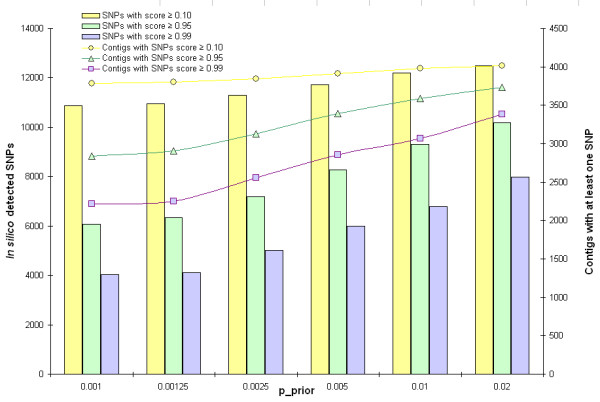
**Number of *in silico *detected SNPs and of snp'ed contigs as a function of the prior probability **. P_prior stands for the *a priori *expected polymorphism rate used by *PolyBayes *to compute the SNP score P_SNP_. A value of p_prior of 0.02 means one SNP expected each 50 nt.

The probability that a position represents a SNP (P_SNP_) depends on the *a priori *expected SNP frequency (termed p-prior in the formula generating P_SNP_) [15]. The p-prior impacts upon the number of SNPs found *a posteriori*; therefore, we tested its influence on the frequency of predicted SNPs (Figure 1). There were fewer predicted SNPs with the lower p-prior values and the impact of the p-prior was greater at higher P_SNP _values. For example, the number of SNPs predicted with P_SNP _≥ 0.10 varied from 12,497 to 10,860 when the p-prior was changed from 0.02 to 0.001, whereas the number of SNPs detected with P_SNP _≥ 0.95 went from 10,173 to 6,085, for p-prior varying from 0.02 to 0.001.

In this report, we used a p-prior of 0.01 corresponding to a mean frequency of one SNP per 100 nucleotides, as in a previous study on *Pinus pinaster *[18]. A very similar SNP frequency was experimentally determined at 0.012 in *Picea glauca*, based upon a total of 9,253 nucleotide sites sampled for eight nuclear genes (*KN1, KN2, KN3, KN4, HB-3, NAM, rpl13A, rpl15*) [21,31]. It is also in the range of that observed for *Pinus taeda *(0.016) [32] and for *Pinus pinaster *(0.0097) [18]. With p-prior = 0.01, we detected, 9,310 SNPs among 3,590 contigs (P_SNP _≥ 0.95; Figure 1), resulting in a polymorphism rate of 1 SNP per 700 nucleotide sites. In *Pinus pinaster*, LeDantec *et al. *(2004) reported a SNP rate of 1 per 660 nucleotide sites by using p-prior = 0.01 and P_SNP _≥ 0.99, and analysing contigs containing 4 reads or more [18]. The rate that we estimated in *P. glauca *became 1 SNP per 689 nucleotide sites, somewhat closer to that reported for *P. pinaster*, when using the same probability levels and considering only contigs of 4 clones or more. Our rate rate further increased to 1 SNP per 492 nucleotide sites when considering SNPs with P_SNP _≥ 0.95 (p-prior of 0.01, contigs with 4 clones). Our analyses also showed that 55.6% to 61.5% the contigs included at least one SNP predicted when varying P_SNP _from ≥ 0.95 to ≥ 0.10 (with a constant p-prior = 0.01: Figure 1).

**Figure 2:**
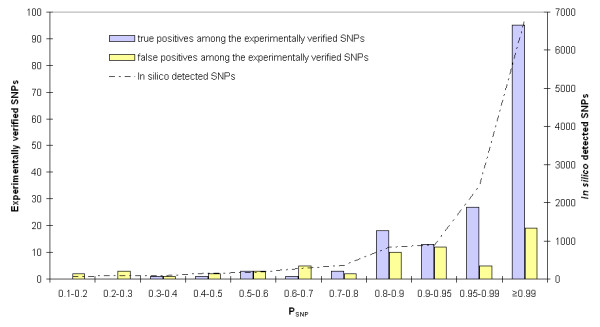
***In silico *detected SNPs and experimentally verified SNPs according to P_SNP _**. A subset of the predicted SNPs was verified by the independant resequencing of fragments amplified from the genomic DNA extracted from the PG653 genotype. The sequence traces were manually inspected to verify the sites where SNPs were predicted by *PolyBayes*. Predicted SNPs that were indeed found in the genomic DNA sequence were called "true positives" (in blue on the figure), whereas the ones that were not verified were called "false positives" (in yellow on the figure).

In our dataset, the 6,459 contigs gave a total of 6,521,041 aligned sites, and 12,264 SNPs were detected *in silico *using *PolyBayes *(minimum P_SNP _= 0). The majority of *in silico *detected SNPs (55.4 %) were detected at P_SNP _≥ 0.99, and most of the remaining SNPs were detected by decreasing the detection stringency to 0.80 (Figure 2, dash line). Further decreasing P_SNP _from 0.80 to 0 added a rather small number of SNPs.

### Experimental estimation of the rate of false positives according to P_SNP_

The collection of *in silico *detected SNPs was built for genotyping purposes. To extract the most reliable SNPs, we could have chosen the ones detected with the highest scores (P_SNP _≥ 0.99). Indeed, several SNP resources were developed by using *PolyBayes *to detect SNPs and only SNP detected with score P_SNP _≥ 0.99 were considered [17,18]. However, the stringent cutoff of P_SNP _≥ 0.99 would eliminate 44.6% of the *in silico *detected SNPs from our data set.(Figure 2). Useche *et al. *(2001) [33] used *PolyBayes *to detect SNPs in maize ESTs. They determined that the P_SNP _score as an important parameter and chose to display all the SNPs in their database, irrespective of their P_SNP _scores, to avoid discarding too many SNPs. However, the validity of the various polymorphisms was not assessed experimentally. We felt it was important to strike a balance between the cost of genotyping and the desire to achieve a high SNP discovery rated, thus, we experimentally tested the *in silico *detected SNPs for various levels of P_SNP _scores.

For the purpose of validation, fragments of spruce genomic DNA were amplified and sequenced from the same source material as the previously conducted EST sequencing [30]. We carried out the analyses using a subset of the dataset, corresponding to the sequences obtained from the single genotype PG653. Working with a single genotype reduced the number of sequencing reactions required for this validation, since detected SNPs represented biallelic positions. Hence, putative SNP sites would correspond to double peaks in the sequence chromatograms derived from PG653 (Figure 2). A set of 156 contigs encompassing 325 SNPs detected *in silico *with a range of P_SNP _values were selected to amplify the genomic sequences. We were able to specifically amplify the single expected sequence for 112 contigs, encompassing 245 *in silico *detected SNPs. A total of 43,286 nucleotides corresponding to these 112 genes were sequenced and were visually inspected by aligning the trace files [see Additional file1] for details about the targeted contigs and SNPs). We obtained a true positive rate of 74%, represented by the *in silico *detected SNPs that were also detected in the genomic DNA sequence analysis (Figure 2). Nearly identical validation rates of 85.1% and 84.9% were observed when considering only the SNPs detected with higher stringency criteria of P_SNP _≥ 0.95 or ≥ 0.99. Based on this observation, we considered SNPs detected with values of P_SNP _≥ 0.95 for the remained of our study. However, the subset of SNPs detected with a P_SNP _≥ 0.99 were used in specific analyses to enable comparisons with published reports [17,18]. Similarly to our results, Le Dantec *et al. *(2004) reported a validation rate of 83% for SNPs detected with a P_SNP _≥ 0.99 in pine [18]. By using a cut-off of P_SNP _≥ 0.95 rather than of P_SNP _≥ 0.99, we increased the number of SNPs by 2,514 out of a total of 12,264 SNPs detected *in silico *using *PolyBayes*.

Such experimental validations of SNPs detected by *PolyBayes *or other computational prediction methods have been conducted only sparingly, and for a few other genomes. *PolyBayes *was used to predict SNPs in human ESTs; a subset of 36 SNPs detected with a P_SNP _score ≥ 0.40 was experimentally verified by screening four populations, leading to a confirmation rate of 56% [15]. On a larger scale, 1,200 SNPs from dbSNP which were detected *in silico *in the human genome by data-mining procedures were experimentally analysed by a pooled DNA sequencing approach, showing that 80% of the detected SNPs were found polymorphic in three ethnic groups [34]. In another validation study, the confirmation rate reached 88%, but the SNPs were detected *in silico *using several approaches and it was not possible to attribute a performance level to *PolyBayes *specifically [35].

We obtained an experimental validation rate 85% (137 out of 161) for SNPs detected in our white spruce contigs at P_SNP _≥ 0.95. The validation rate increased to 92% when only considering contigs derived from 2 or 3 clones. The SNPs falsely predicted by *PolyBayes *represent either experimental errors in the ESTs or differences between paralogous sequences erroneously assembled into a single contig. The experimental error rate was previously shown to be very low, from the analysis of overlapping sequence reads in opposite directions from the same clone. On the other hand, the higher confirmation rate observed with contigs containing fewer clones would indicate that putative paralogs are indeed present in the contigs, especially in those comprised of several cDNA clones, in spite of the stringency of the parameters used in the contig assembly. When the complete genome is available to help delineate orthologs and paralogs, the confirmation rate of *in silico *detected SNPs tends to be higher than that observed in our study. For example, in a SNP detection project conducted in *Arabidopsis*, the confirmation rate was 96% for *in silico *detected SNPs [5]. Analysing SNPs in a completely sequenced genome can be much more powerful because most of the paralogs may be excluded from the contigs used to detect SNPs. Our results confirm the need for experimental validation of *in silico *SNP detection in genomes that are incompletely sequenced, before the information is used for more advanced genetic analyses. In the following section, we examine two methods to estimate the proportion of paralogous SNPs among *in silico *detected SNPs and show that most of the SNPs falsely predicted by *PolyBayes *represent differences between paralogous sequences.

### Estimating the proportion of paralogous SNPs

A number of contigs were likely to contain paralogous sequences, so we were interested in evaluating the proportion of paralogous versus orthologous SNPs in the dataset. We use the term 'paralogous SNPs' to refer to non allelic SNPs which likely result from the misassembly of paralogous, and 'orthologous SNPs' for allelic differences occurring between reads truly derived from the same gene. The paralogous SNPs are to a great extent undesirable for population or association genetics studies and in genetic mapping. To estimate the proportion of paralogous SNPs, we followed two approaches.

We first used the previous validation experiment of *in silico *detected SNPs where a set of 112 contigs was re-sequenced from genomic DNA of the single genotype PG653. The same set of contigs was re-sequenced from genomic DNA of a haploid white spruce megagametophyte, with 43,286 nucleotides determined and visually inspected by aligning the trace files. The presence of superimposed peaks in sequences from haploid DNA indicates the presence of 'paralogous SNPs' We found 19 SNPs (out 161) that corresponded to multiple peaks in the sequences obtained from haploid megagametophyte DNA, among the *in silico *detected SNPs used for validation, with genomic DNA of PG653 (see above), which represents a proportion of paralogous SNPs of 12%. If we only consider the SNPs detected *in silico *with P_SNP _≥ 0.95, the proportion of paralogous SNPs decreases slightly to 10.5%.

The second method used is based on population genetics theory and relies entirely on estimates of *in silico *SNP frequencies, as described in Methods. It relies on a comparison of the frequency of SNPs in contig sequences derived only from the single genotype PG653, and the frequency of SNPs in the total population sampled. When paralogous sequences are intermixed with orthologous sequences, the proportion of segregating sites *k *is biased upward by a quantity corresponding to ε. ε is a constant independent of the number of genotypes analysed, while *k *will increase with the number of haploid genomes analysed, thus resulting in a reduction of ε relative to *k*. This principle can be used to estimate ε. For this purpose, only SNPs with P_SNP _≥ 0.95 and contigs containing 10 clones or more were considered, in order to sample as many distinct haploid genomes as possible while sampling a reasonable number of contigs and SNPs. When sequences corresponding to contigs obtained from PG653 only were considered, 43 SNPs were detected from a cumulative length of 36,191 nucleotide sites, for a *k *value of 1/842 or 0.00119. When sequences from all genotypes were considered for the same contigs, 1,532 SNPs were detected from a cumulative length of 507,528 nucleotide sites, and *k *was higher with a value of 1/331 or 0.00302. Then, the estimated proportion of «paralogous SNPs» in the dataset, ε, was obtained by solving the set of equations (4) and (5) in Methods and for two sampling scenarios delineated therein. The ε values obtained were 0.000238 and 0.000517, respectively, corresponding to proportions of paralogous SNPs of 8% and 17% in the entire dataset. As explained in Methods, the second scenario, which assumes a sampling size of 24 haploid genomes, is less likely than the first scenario of sampling 10 distinct haploid genomes per contig, given that very few contigs would be characterized by a number of distinct clones approaching 24, the maximum number of haploid genomes corresponding to the 12 distinct genotypes used to obtain EST sequences. Thus, the value of 8% should be more realistic than the value of 17%.

These ε estimates agree well with the proportion of paralogous SNPs obtained from experimental validation using haploid megagametophyte DNA (rate of 10 to 12%). These estimates indicate that most of the false positives predicted by *PolyBayes *were not experimental errors, but that the criteria used to assemble the ESTs and run *PolyBayes *were stringent enough to avoid a wide occurrence of paralogous SNPs. To our knowledge, these are the first experimental and statistical estimates of proportions of paralogous SNPs in SNP datasets derived from EST contigs. The statistical procedure outlined above offers a simple way to estimate the proportion of paralogous SNPs for a given set of contig assembly parameters, as long as EST sequences from a single genotype can be traced back. However, this procedure may not be suitable when the reference genotype has been genetically manipulated.

### SNP frequency according to contig depth and length

**Figure 3:**
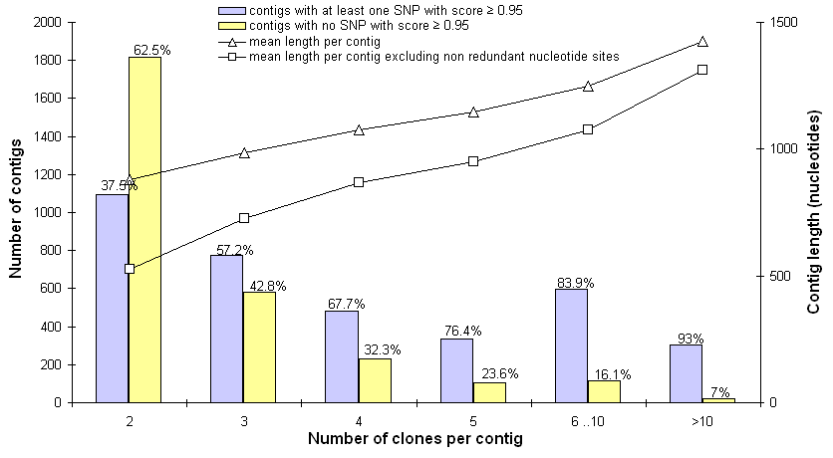
**Number of contigs including *in silico *SNPs detected with P_SNP _≥ 0.95 **. Mean size of the contigs according to the length of the consensus sequence or mean size of the alignment per contig according to the number of clones.

Several studies reported SNP frequencies from automated detection methods, though SNP frequencies can vary according to several sampling parameters. In the next analysis, we considered the effects of contig depth, contig length, and alignment length on the estimates of SNP frequency from *in silico *detection with *PolyBayes *(Table 1, Figure 3). The number of contigs with or without SNPs was counted according to the number of clones within each contig, which defines the contig depth. The proportion of contigs that contained at least one SNP (P_SNP _≥ 0.95) increased from 37.5 % to 93%, for contigs derived from 2 and 10 clones respectively. (Figure 3). The relative frequency of SNPs also increased asymptotically according to the contig depth (Table 1), as was reported for *Pinus pinaster *[18]. The larger number of SNPs detected resulted from the increase of both the depth of the contigs and the length of the alignments (Figure 3, Table 1).

To evaluate SNP frequencies, two methods were used (Table 1). First, the number of *in silico *detected SNPs was divided by the cumulative length of the contigs, resulting in a number of SNPs per nucleotide site (P_SNP _≥ 0.95). The SNP frequency ranged from one per 1,184 nucleotide to one per 361 nucleotide sites in contigs from two to 10 or more clones, respectively (Table 1). On average, one SNP was detected per 700 nucleotide sites. Second, we generated a less biased measure of the SNP frequency by computing the number of SNPs (P_SNP _≥ 0.95) per redundant nucleotide site. A redundant site is one where information for more than one sequence is available in the contig. We thus developed a Perl script to only count the number of redundant nucleotide sites involved in the SNP detection within each contig, thus excluding non redundant nucleotide sites, which were only represented by one sequence in the contig alignment. With this approach, the SNP frequency range from one per 712 redundant nucleotide to one per 331 redundant nucleotide sites for contigs of two or 10 and more clones, respectively. We thus detected one SNP per 511 redundant nucleotide sites, across all the contigs with 2 or more clones (Table 1). These estimates of SNP frequency have less of a downward bias than estimates based on the total alignment length because non redundant nucleotide sites are excluded. The difference in mean alignment length per contig can be seen on Figure 3 and Table 1, when excluding or including non redundant nucleotide sites. As contigs contain more clones, the relative impact of this factor diminishes (Figure 3). The proportion of paralogous SNPs estimated in the previous section was also based on SNP rates per redundant nucleotide site.

### Detection of putative coding regions by sequence similarity searches and ORF prediction

To determine which SNPs lay outside or inside putative coding regions, two methods were used to predict open reading frames (ORFs). The first one was based on similarities with protein sequences in public databases and the second one was based on *ab initio *predictions of open reading frames.

In the first approach the *blastx *program was used to compare translations of the spruce contigs with protein sequences from the Uniref100 database and the *Arabidopsis *TAIR database. The alignments with homologous sequences at the protein level enabled us to localize conserved coding regions and to determine the frame of the coding sequences within boundaries defined by sequence conservation. If a SNP site lay within the boundaries of the alignment, then it was considered as belonging to a coding region conserved across species. If the SNP lay outside of these boundaries, we could not characterize the nature of the SNP. Out of the 3,590 contigs containing SNPs (P_SNP _≥ 0.95), 3,160 contigs had a match with an *Arabidopsis *protein or a protein from Uniref100 with *blastx *(e-value < 1e-10; Table 2). The remaining 430 orphan contigs containing SNPs did not have strong enough alignments to draw conclusions.

A second method based on ORF prediction was used to pursue two objectives. It enabled us to identify putative ORFs among the 430 orphan contigs and to classify the SNPs as coding or not coding without any assumptions regarding sequence similarity. ORFs were predicted by using the *Diogenes *software which was trained with ORFs from several species, thus generating multiple matrices to detect ORFs in plant sequence datasets [36]. We analysed the results generated by two matrices, one derived from the "Brassicaceae" and one from the "Pinaceae" training sets, using a p-value < 1e-08 in each case (Table 2). *Diogenes *detected ORFs with a in 214 out of the 430 orphan contigs (p-value < 1e-08). with only 9 detected by *Diogenes *trained with Pinaceae sequences only. We combined the ORFs detected by *blastx *and *Diogenes *and we were able to localize an ORF for a total of 3,374 out of the 3,590 snp'ed contigs. An ORF was assigned to 88% of the contigs based on *blastx *matches alone, whereas the combination of both *blast *and *Diogenes *increased the assignments to 94%. The assignments were distributed as follows: i) 3,160 ORFs with a *blastx *hit in Uniref100 (including 20 sequences with a match in the *Arabidopsis *proteome but not in UniRef100); ii) 225 ORFs predicted by *Diogenes*-Brassiceae; and iii) 9 ORFs specifically predicted by *Diogenes*-Pinaceae.

### Estimating the rates of nonsynonymous and synonymous SNPs

In order to classify synonymous and nonsynonymous SNPs, we used the boundaries of the ORFs predicted by *Diogenes *wherever possible using, in preference over the boundaries of the conserved regions detected by *blastx*. Boundaries of coding regions delineated by similarity searches (*blastx*) were avoided because they could have introduced an estimation bias related to conserved regions. We compared the *blastx *and the *Diogenes *results to annotate the coding regions based on ORF predictions and developed and improved dataset to classify nonsynonymous and synonymous SNPs and estimate their frequencies. The improved dataset was still comprised of 3,374 ORFs but distributed as follows: i) 2,823 ORFs predicted by *Diogenes*-Brassiceae ii) 9 ORFs specifically predicted by *Diogenes*-Pinaceae, iii) 533 regions similar to known proteins identified by *blastx *searches against Uniref100 but without any ORF predicted by *Diogenes*, and iv) 9 regions similar to *Arabidopsis *proteins identified with *blastx *but without any ORF predicted with *Diogenes*.

We thus classified 3,789 coding SNPs, distributed into 39.8% nonsynonymous and 60.2% synonymous substitutions (Table 3). Whatever the method used to delineate the ORFs (*blastx *or *Diogenes*), the ratio of synonymous to nonsynonymous substitutions was about 1.5:1. This ratio is lower than the ratio of 2:1 found in *Arabidopsis *by Schmid et al. (2003) [5], based on the sampling of 5,289 contigs. However, these numbers should not be compared directly because they are not standardized by the numbers of synonymous and nonsynonymous sites sampled. To estimate the overall rates of synonymous and nonsynonymous SNPs per nucleotide site, the numbers of synonymous (*Ls*) and nonsynonymous sites (*La*) [37] were determined based on the ORFs predicted as described above. Based on these 3,374 ORFs, the overall rate of synonymous substitutions was 5.18 SNPs per 1,000 sites and the overall rate of nonsynonymous substitutions was 0.89 SNP per 1,000 sites (Table 3). Compared to direct count estimates, the difference between the two classes of SNPs was increased because there exists far fewer synonymous sites than nonsynonymous sites. The ratio of nonsynonymous to synonymous SNP rates per site (*Ka*/*Ks*) was 0.17. Such a ratio far below 1 indicates that, on average, white spruce ORFs are under strong purifying selection. Using the ORFs delineated by the *blastx *search or predicted by *Diogenes *had no effect on the estimation of SNP frequencies, indicating that there was no bias associated with the delineation of coding regions using *blastx*. Similar *Ka*/*Ks *ratios based on smaller numbers of genes have been estimated in *Arabidopsis*. The white spruce "genome-wide" *Ka*/*Ks *ratio (0.17) is identical to the mean *Ka*/*Ks *of 0.17 estimated from 23 nuclear genes in *Arabidopsis *[38], and it is similar to the mean *Ka*/*Ks *ratio of 0.207 obtained from 242 genes in *A. thaliana *[39]. Several studies have found a large gene-to-gene variance in *Ka*/*Ks *values. Similar variation was also found in the present study (data not shown).

By combining several approaches to delineate ORFs we were able to increase the number of coding SNPs by 13%. With *Diogenes*, we classify 221 SNPs (among the 430) contigs with no match in Uniref100. In this subset of 221 SNPs, the rate of synonymous substitutions was 6.18 SNPs per 1,000 sites and that of nonsynonymous substitutions was 1.10 SNP per 1,000 sites, which are slightly higher rates than the overall dataset., but the overall *Ka*/*Ks *ratio remained about the same at 0.18. This result was expected, as the discovery of additional SNPs by *in silico *methods should not affect the overall balance between nonsynonymous and synonymous SNPs.

### Integration of the SNP resource in ForestTreeDB

To facilitate the retrieval of snp'ed contigs according to their functional annotations, the white spruce SNP data was uploaded into ForestTreeDB, a database unifying EST data and sequence annotations. ForestTreeDB includes tables containing data related to 12,264 white spruce SNPs detected *in silico*. These public query pages are available to mine the data at . Each SNP is described by its location on the contig sequence, including the appropriate strand. The possible bases of the SNP are represented by the IUPAC nucleotide ambiguity code. An "evidence code" indicates the SNP status as "predicted", "validated," or "not validated". The number of distinct clones that support each SNP location is retained, as well as the analytical parameters used for the prediction, including the p_prior value and the confidence level of the *in silico *detection (P_SNP_). Several computational approaches were used to explore the putative functions of the sequences recovered in the snp'ed contig dataset the results were incorporated in the ForestTreeDB database. The analyses included sequence similarity searches against several databases and Hidden Markov Models searches with the models available in the PFAM protein families database, as described previously [30]. Approximately 87% of the contigs containing at least one SNP had a *blastx *hit in UniRef100 (e-value < 1e-10; Table 2). We assigned putative GO terms to the subset of snp'ed contigs through the sequence similarity searches against UniRef100 and the *Arabidopsis *TAIR database providing a link to molecular function terms in the Gene Ontology (GO). Among the diverse molecular function categories, 1,943 annotations (belonging to 94 functional classes) were associated to 1,388 snp'ed contigs (one contig may be associated to more than one GO term). The wide diversity of predicted functions among the snp'ed contigs. indicates that dataset could be useful in diverse association studies drawing upon genes from diverse protein functions.

**Figure 4:**
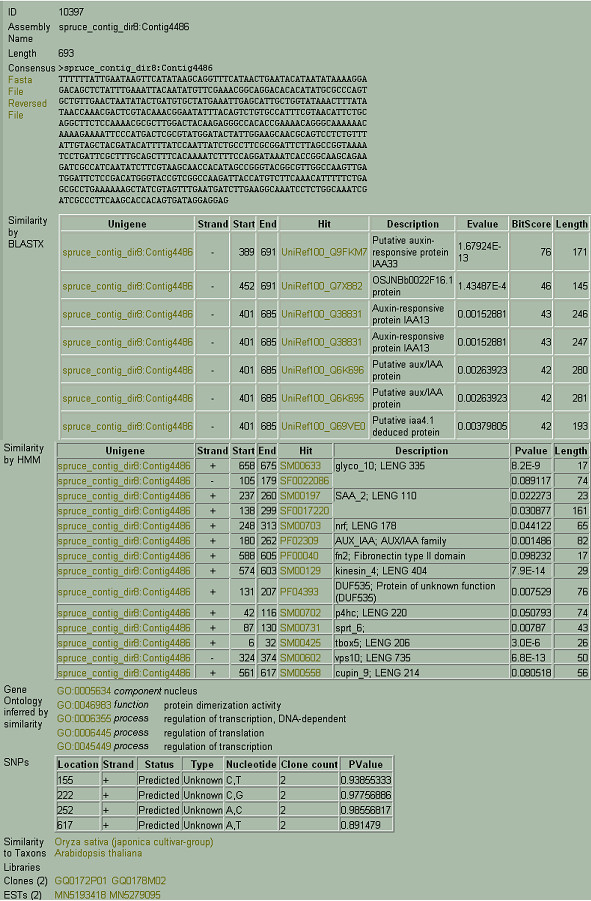
**ForestTreeDB screenshot **showing the result from a query based on the Contig4486 (ID: 10387). This page displays the Gene Ontology terms associated to the contig and SNP data and the similarity data obtained by Hidden Markov Model searches against the domains and families available in the PFAM  and SMART  database. A SNP table displays four SNPs predicted by *PolyBayes *in Contig4486, with P_SNP _scores ranging from 0.89 to 0.98. Links also allow retrieval of the members (clones and ESTs) of the studied contig, their sequences, as well as the read alignment in a MSF format.

Among other applications, ForestTreeDB is particularly useful in the context of candidate gene approaches, as it provides *a priori *information on predicted gene polymorphisms along with predicted protein functions. ForestTreeDB interfaces enable researchers to visualize and retrieve sequences, contig compositions, sequence similarity search results and prediction by *PolyBayes *in a parsed format. Figure 4 provides an example based on the Contig4486: it is similar to a sequence belonging to the auxin-responsive IAA family, is classified in the "transcriptional regulation" Gene Ontology category and encompasses four SNPs. Such integration of SNP data into ForestTreeDB will facilitate the selection of the most promising SNPs to be incorporated in genetic analyses either based on their reliability (probability score, depth of the EST alignment) or based on the functional annotation of the sequences.

## Methods

### Plant material and EST collection

A total of 17 *Picea glauca *cDNA libraries were prepared from a diverse tissues sampled to maximize the diversity of the isolated genes. Details about the sampled tissues are available on the Arborea web site [29]. Nine libraries were prepared from the genotype PG653, and the remaining eight libraries were prepared from 11 different accessions of *Picea glauca *to incorporate more genotype diversity. Details of the preparation of cDNA libraries and EST sequencing methods previously presented elsewhere [30].

All of the clones were randomly chosen and sequenced from the 3' end [30]. A subset of clones selected among 10 libraries were also sequenced from the 5' end. As the 3' and 5' reads from the same clone may overlap in the same contig, all SNP diversity and distribution parameters were estimated by considering the number of clones analysed instead of the number of reads. Thus, we directed our search for SNPs to the 6,459 contigs derived from at least 2 clones and which had mean length 1,009 nucleotides of *Phred *score above 20. The majority of contigs, namely 73.4%, were derived from both 3' and 5' reads; they had an average length of 1,078 nucleotide and represented completely sequenced inserts. A total 20.4% of the contigs were derived from the 3' reads and only 6.2% were from the 5' reads alone. The 5' and 3'contigs were slightly shorter, with mean lengths 803 and 823 nucleotides, respectively. The EST sequences were deposited in the dbEST section of the GenBank database. The contig sequences, the contig composition, *blast *reports, and some statistics about the sequence dataset are available at our web site [28].

### Experimental validation of the *in silico *detected SNPs by resequencing of genomic DNA

Experimental validation of the *in silico *detected SNPs involved PCR amplification and sequencing of genomic DNA corresponding to EST contigs. To minimize the risk of amplifying multiple members of the same multigenic family when amplifying genomic DNA corresponding to EST contigs, we chose contigs with a small number of hits against the more extensively sequenced pine transcriptome (Pinus Gene Index Release 4)[26]. We assumed that these contigs would be part of small gene families and would also be less represented in the spruce genome. We also extrapolated the ATG and/or the STOP positions on the spruce contigs based on the assumption that spruce proteins would be of similar size than the complete *Arabidopsis *proteins against which they had a match. We thus anchored one of the two primers (or both) upstream of the ATG or downstream of the STOP codons to target the less conserved untranslated regions. For the contigs in which ATG or STOP codons could not be predicted, we selected sequence regions that were not conserved with known sequences to anchor at least one of the two PCR primers. Primers for PCR amplification and sequencing were designed by using the *Primer3 *software [40] installed locally. The primer sequences were compared with *blast *[41] against the spruce and the pine transcript datasets to exclude primers with possible hits in multiple transcripts. PCR reactions were performed in 30 μl containing 20 mM Tris-HCl (pH 8.4), 50 mM KCl, 1.5–2.0 mM MgCl_2_, 200 μM of each dNTP, 200 μM of both 5' and 3' primers and 1.0 unit of Platinum *Taq *DNA Polymerase (Invitrogen, Carlsbad, California). About 5–20 ng of genomic DNA was used as template. A peltier Thermal Cycler (DNA Engine, DYAD™, MJ Research, Walthman, Massachusetts) was used, with the following thermal cycling program : 4 min at 94°C, followed by 35 cycles of 30 s at 94°C, 30 s at annealing temperature optimized between 54 and 60°C for each pair of primers, and 1 min at 72°C, followed by 10 min at 72°C. Each PCR product was directly sequenced in both directions with a Perkin-Elmer ABI 3700 XL DNA sequencer (Applied Biosystems, Foster City, California), using BigDye Terminator cycle sequencing kit (version 3.1). Contigs were constructed with the *seqmerge *program in the GCG package (Wisconsin Package Version 10.3, Accelrys Inc., San Diego, California).

### EST sequence processing and SNP automated detection

EST sequences were processed with locally-developed system at the Center for Computational Genomics and Bioinformatics, University of Minnesota, for contig quality control, as was previously described in detail [30]. Sequence trace files from the spruce ESTs were processed to yield raw sequences with the *Phred *base calling software version 0.020425.c [42]. *Phred *quality values less than 20 were considered to be ambiguous in this experiment and were assigned base N. Quality trimming and vector filtering (with polyA/polyT removal, as appropriate) were done using the software *gstVF4 *[43]. Processed EST sequences were then assembled using *Phrap *(version 0.990319) [44]. *Phrap *contigs were evaluated for chimeric sequences, and reassembled so as to minimize chimeric tendencies. Final assembly threshold for phrap were -minmatch 50 -minscore 100. This procedure produced better quality contigs prior to running *PolyBayes*. For the SNP prediction, the *PolyBayes *software version 3.0 [15] was run with the following parameters: -inputFormat ace -aceIn spruce.fasta.screen.ace.1 -phdFilePathIn../phd_dir -reportOut../polybayes.out -p_prior (0.001 0.00125 0.0025 0.005 0.01 0.02) -thresholdSnp 0. The candidate SNPs were filtered by using the probability P_SNP _provided by *PolyBayes *with several cutoffs for P_SNP_. *PolyBayes *outputs were analysed with in-house scripts written in Perl.

### Statistical estimation of the proportion of paralogous SNPs

The following procedure was used to estimate the proportion of paralogous versus orthologous SNPs in the SNP dataset. In the absence of paralogous SNPs, it can be shown that

*k *= *a*θ     (1)

where *k *is the proportion of segregating sites, those harboring a SNP, and θ = 4*N*_*e*_μ, *N*_*e *_being the effective population size and μ, the mutation rate per site per generation, and

*a *= 1 + 1/2 + ... + 1/(*n*-1)     (2)

where *n *is the number of sequences sampled per gene (eq. 9.8, [45]). When paralogous sequences are inter-mixed with orthologous sequences, *k *is biased upward by a quantity corresponding to ε, such that

*k *= *a*θ + ε     (3)

Thus, ε could be estimated by solving the following set of equations:

*k*_PG653 _= θ + ε     (4)

*k*_pop _= *a*θ + ε     (5)

where *k*_PG653 _and *k*_pop _correspond to the number of segregating sites in sequences from the single genotype PG653 and from the population sample of 12 genotypes, respectively. In both cases, the number of sites sampled corresponded to the cumulative length of sequences of redundant nucleotide sites, those represented in at least two clones, and we used only SNPs with P_SNP _≥ 0.95. Because contigs with a small number of clones are biased downward in terms of sampling SNP diversity (Figure 3), we limited our analysis to contigs containing 10 clones or more when considering sequences from PG653 only as well as when considering sequences from all 12 genotypes. In the case of PG653 and equation (4), we assumed that all contigs with 10 clones and more contained sequences from both haploid complements, thus the sampling size *n *corresponded to 2 and thus, *a *= 1 was used when transforming equation (1) to (4). When considering all 12 genotypes and equation (5), lower and higher bound scenarios were considered. A first scenario assumed a sampling size of 10, given that contigs with 10 or more sequences were analysed, thus corresponding to an *a *value of 3.733 in equation (5). We also tested a second scenario, by assuming a sampling size of 24 corresponding to the number of haploid complements used to obtain EST sequences, with corresponding *a *value of 2.929 in equation (5). This scenario is less likely, given that very few contigs had a number of sequences in excess of 30. Then, the proportion of paralogous SNPs in the entire dataset could simply be obtained by the ratio of ε ove*r k*_pop_.

### ORF annotation

*Phrap *contigs were analysed by *blastx *comparisons [41] against several databases, including a non-redundant peptide set (UniRef100) provided by the UniProt consortium [46], *Arabidopsis *protein dataset retrieved from the TAIR web site [3], and the Pine Gene Index (PGI4) retrieved from TIGR [26]. We extrapolated the Gene Ontology (GO) terms [47,48] associated with sequences in UniRef100 and the TAIR databases to the contigs using sequence similarity criteria. The *Diogenes *software [36], which was trained with sequences from multiple species to predict open reading frames, was used with two parameter sets derived respectively from Brassicaceae and Pinaceae species. Only those predicted coding regions with p < 1e-08 were retained. A Perl script was written to standardize the *blastx *output and the *Diogenes *output (frames were converted into phases, and the start/end of the ORFs were recalculated to comply with the *blast *HSP start/end). To sort the SNPs into coding and non coding classes, a Perl program was developed to extract from the contig sequence the coding regions, to split them into triplets based on the frame information and then to deduce the codons based on the strand information. To determine whether the SNP induced an amino acid change, the CodonTable from Bioperl was incorporated into the main script.

## Authors' contributions

NP: coordination of bioinformatics activities, data analysis, preparation of the manuscript; LP: data analysis, EST assembly, programming; CP: sequence annotation, gene ontology assignment, databasing; JM: supervision and management of the sequencing project, preparation of the manuscript; JB: supervision of the SNP project, data analysis, preparation of the manuscript. All authors approved the manuscript.

## Supplementary Material

Additional file 1 **Experimentally verified SNPs**. Locations and sequences of the PCR primers, SNP location and prediction scores, presence of SNPs in the sequenced PCR fragments, functional annotation of the contigs. Click here for file

## Figures and Tables

**Table 1:** Frequency of detected SNPs with P_SNP_score ≥ 0.95 in the contigs derived from all 12 genotypes used to obtain ESTs

Number of clones in the contig	Number of contigs	SNPs	Cumulated length of the contigs	SNPs/nucleotide site^1^	Cumulated length of the contigs excluding non redundant sites^1^	SNPs/redundant nucleotide site^2^
2	2911	2,158	2,554,730	1:1,184	1,536,400	1:712
3	1356	1,814	1,337,593	1:737	987,177	1:544
4	715	1,196	769,584	1:643	620,190	1:518
5	441	915	504,813	1:552	419,567	1:458
6	284	695	345,924	1:498	293,883	1:422
7	159	375	202,507	1:540	169,052	1:451
8	119	358	150,187	1:419	130,071	1:363
9	81	267	101,714	1:381	91,286	1:342
≥10	393	1,532	553,392	1:361	507,528	1:331
Total	6459	9,310	6,521,041	1:700	4,755,154	1:511

^1 ^Sites of the contigs where only one sequence has been determined.

^2 ^Sites of the contigs where more than one sequence has been determined.

**Table 2:** The number of contigs with identifiable coding regions. ORFs were delineated based on one method (on the diagonal), based on a combination of two methods (in bold), and based on data found by both methods (in italics). The total number of snp'ed contigs was 3,590 (P_SNP_≥ 0.95).

Method	Blastx against uniprot e-value < e-10	Blastx against Arabidopsis e-value < e-10	Diogenes Brassicaceae-trained p-value < e-08	Diogenes Pinaceae-trained p-value < e-08
Blastx Uniprot e-value < e-10	3140			
Blastx Arabidopsis e-value < e-10	**3160 ** *3060*	3080		
Diogenes Brassicaceae-trained p-value < e-08	**3356 ** *2607*	**3338 ** *2565*	2823	
Diogenes Pinaceae-trained p-value < e-08	**3276 ** *1929*	**3256 ** *1889*	**2874 ** *2014*	2065

**Table 3:** Descriptive parameters of coding SNPs

Parameter	Blastx/Uniprot proteins e-value < 1e-10	Blastx/*Arabidopsis *proteins e-value < 1e-10	Diogenes ORF Brassicaceae trained p-value < 1e-08	Diogenes ORF Pinaceae trained p-value < 1e-08	Dataset of 205 ORF predicted by Diogenes but with no match in Uniprot e-value < 1e-10	Combination of all methods
Contigs with a putative coding sequence assigned	3,140	3,080	2,823	2,065	205	3,374
Contigs with no coding region assigned	450	510	767	1,525	-	196
Unclassified SNPs	3,910	3,853	3,202	2,626		3,923
Synonymous SNPs (1)	2,013	1,951	2,072	1,468	132	2,282
Nonsynonymous SNPs (2)	1,339	1,309	1,347	972	89	1,507
Total coding SNPs	3,352	3,260	3,419	2,440	221	3,789
synonymous/nonsynonymous SNPs	1.50	1.49	1.54	1.51	1.48	1.51
Number of nonsynonymous sites (*La*)	1,529,942.94	1,493,852.65	1,501,524.04	1,060,194.41	80,471.00	1676414.38
Number of synonymous sites (*Ls*)	401,769.06	391,332.35	393,089.96	277,718.59	21339.01	440352.62
Total number of coding sites (*La*+*Ls*)	1,931,712	1,885,185	1,894,614	1,337,913	101,811	2116767
Rate of nonsynonymous SNP per site (2)/*La *(*Ka*)	0.00087	0.00087	0.00090	0.00092	0.00110	0.00089
Rate of synonymous SNP per site (1)/*Ls *(*Ks*)	0.00501	0.00498	0.00527	0.00528	0.0062	0.00518
Ratio *Ka/Ks*	0.174	0.175	0.170	0.174	0.179	0.172
